# Dissemination of clinical *Escherichia coli* strains harboring *mcr-1*, *bla*_NDM−7_ and siderophore-producing plasmids in a Chinese hospital

**DOI:** 10.1186/s13756-024-01423-3

**Published:** 2024-06-18

**Authors:** Lihua Liu, Mingqi Zhao, Yanhua Tang, Aihua Shen, Xiao Yang, Li Yao, Dongxing Tian

**Affiliations:** 1 Department of Clinical Laboratory, Affiliated Hospital of Jining Medical University, Jining, Shandong Province China; 2 Affiliated Hospital of Jining Medical University, Jining, Shandong Province China; 3 Department of Respiratory and Critical Care Medicine, Affiliated Hospital of Jining Medical University, Jining, Shandong Province China; 4 Department of Clinical Laboratory, Jining Wenshang Hospital, Jining, Shandong Province China; 5 Postdoctoral Mobile Station of Shandong University of Traditional Chinese Medicine, Jinan, Shandong Province China

**Keywords:** Carbapenem-resistant *Enterobacterales*, Mcr-1, blaNDM-7, Siderophore-producing plasmids, *Escherichia coli*

## Abstract

**Background:**

Carbapenem-resistant *E. coli* (CREco) pose a significant public health threat due to their multidrug resistance. Colistin is often a last-resort treatment against CREco; however, the emergence of colistin resistance gene *mcr-1* complicates treatment options.

**Methods:**

Two *E. coli* strains (ECO20 and ECO21), recovered from hospitalized patients in distinct wards, exhibited resistance to carbapenems and colistin. Whole-genome sequencing and phenotypic characterization were employed to study resistance patterns, plasmid profiles, transferability of resistance and virulence genes, and siderophore production capabilities. Comparative genome analysis was used to investigate the genetic environment of *mcr-1*, *bla*_NDM−7_, and virulence clusters.

**Results:**

Both *E. coli* strains exhibited thr presence of both *mcr-1* and *bla*_NDM−7_ genes, showing high resistance to multiple antibiotics. Genomic analysis revealed the clonal transmission of these strains, possessing identical plasmid profiles (pMCR, pNDM, and pVir) associated with colistin resistance, carbapenem resistance, and virulence factors. Conjugation experiments confirmed the transferability of these plasmids, indicating their potential to disseminate resistance and virulence traits to other strains. Comparative genomic analyses unveiled the distribution of *mcr-1* (IncX4-type) and *bla*_NDM_ (IncX3-type) plasmids across diverse bacterial species, emphasizing their adaptability and threat. The novelty of pVir indicates its potential role in driving the evolution of highly adaptable and pathogenic strains.

**Conclusions:**

Our findings underscore the co-occurrence of *mcr-1*, *bla*_NDM−7_, and siderophore-producing plasmids in *E. coli*, which poses a significant concern for global health. This research is crucial to unravel the complex mechanisms governing plasmid transfer and recombination and to devise robust strategies to control their spread in healthcare settings.

**Supplementary Information:**

The online version contains supplementary material available at 10.1186/s13756-024-01423-3.

## Introduction

Carbapenem-resistant *Enterobacterales* (CRE), known for their multidrug resistance or extensively drug-resistant profiles, pose a severe threat to public health due to their association with high mortality and high transmissibility [1, 2]. With limited treatment options for CRE infections, colistin—an essential cationic antimicrobial peptide with neurotoxic effects—is considered the final resort against CRE [3]. However, the emergence and global dissemination of the plasmid-borne colistin resistance gene, *mcr-1*, presents a looming clinical crisis, potentially leading to a scarcity of effective antibiotics [4].

Carbapenemases serve as the primary cause of carbapenem resistance in CRE. KPC enzymes commonly prevail in *Klebsiella pneumoniae*, whereas NDM enzymes predominate in *Escherichia coli* [5]. Beyond their detection in hospitalized patients and healthy individuals, these enzymes are increasingly prevalent in diverse environments, including poultry, pets, water sources, and clinical settings [6, 7]. The rapid discovery of over 60 NDM variants since the identification of NDM-1 in India (https://www.ncbi.nlm.nih.gov/pathogens/refgene/#NDM), suggests their adaptability, possibly fostering escalation among humans and food-producing animals. Similarly, since the first transferable *mcr-1* was found in *E. coli* from China (2016), various *mcr* variants (*mcr-1* to *mcr-10*) have been reported globally in diverse multidrug-resistant *Enterobacterales*, with *mcr-1* emerging as the most prevalent variant [8]. Iron, crucial for bacterial growth and reproduction, prompts certain bacteria to secrete high-affinity siderophores like aerobactin and salmochelin, aiding their growth and pathogenicity [9, 10]. Notably, aerobactin genes *iucABCD-iutA*, residing on virulence plasmids, are considered pivotal pathogenic factors in *K. pneumoniae* [9], although reports of such plasmids in *E. coli* are relatively infrequent.

Plasmids, serving as carriers for genes like *mcr-1*, *bla*_NDM_, and the virulence factor *iucABCD-iutA*, pose a significant threat by facilitating their transmission among humans, animals, and the environment. This dissemination could culminate in larger, potentially uncontrollable global outbreaks, amplifying clinical treatment pressures. Therefore, the imperative is the control of plasmid spread at its source to manage carbapenem-resistant *E. coli*, colistin-resistant *E. coli*, and siderophore-producing *E. coli*.

*E. coli* concurrently carrying *mcr-1*, *bla*_NDM_, and *iucABCD-iutA* genes possess the potential to confer carbapenem, polymyxin resistance, and siderophore production, paving the way for the emergence of more menacing pathogens. Our study, isolating two strains of *E. coli* from hospital environments bearing *mcr*-positive, *bla*_NDM_-positive, and virulence plasmids, underscores their self-transmissible nature, capable of transferring both resistance and pathogenicity individually or simultaneously to other strains. Immediate measures to control the spread of such strains are imperative.

## Materials and methods

### Bacterial strains

Two clinical *Escherichia coli* strains (ECO20 and ECO21) which were resistant to carbapenems and colistin, were identified from previous daily work and were stored at − 80 °C in 10% glycerol. After subculture on blood agar plates for 24 h, isolates were identified by MALDITOF/MS (matrix-assisted laser desorption/ionization time-of-flight mass spectrometry). Carbapenem resistance genes and colistin genes were detected by amplification and sequencing. Detailed anonymized metadata, such as patients’ gender, age, dates of sample collection, sample types, and hospitalization days, were recorded for analysis.

### Antimicrobial susceptibility testing

We conducted antimicrobial susceptibility testing by the broth microdilution method using 0.5 McFarland inoculum suspensions diluted 1:100 to a final concentration of 10^6^ CFU/ml. Ampicillin (AMP), ampicillin/sulbactam (SAM), piperacillin/tazobactam (TZP), cefazolin (CZO), cefuroxime (CXM), ceftriaxone (CRO), cefepime (FEP), cefoperazone/sulbactam (CSL), gentamicin (GEN), levofloxacin (LVX), trimethoprim/sulphamethoxazole (SXT), imipenem (IPM), meropenem (MEM), tigecycline (TGC), nitrofurantoin (NIT), amikacin (AMK), and colistin (COL) were selected to determine the minimum inhibitory concentrations (MICs). MICs were defined as the lowest concentrations of antimicrobials in the wells where no visible bacterial growth was observed. *E. coli* ATCC 25,922 served as a standard reference strain.

### Compete genome sequencing and bioinformatics analysis

The genomic DNA of ECO20 and ECO21 isolates were extracted using the bacteria DNA isolation mini kit (Tiangen, Shanghai, China). Combined Oxford Nanopore (MinION system, Nanopore) and Illumina sequencing (NovaSeq system, Illumina Inc) were used to obtain complete chromosome and plasmid sequences. *De novo* assembly was performed using HGAP and CANU using default settings [11, 12]. The multilocus sequence type, serotype, and fimtype were determined using Center for Genomic Epidemiology (CGE) web tool (https://www.genomicepidemiology.org/services/). The virulence genes were identified using VFDB (http://www.mgc.ac.cn/cgi-bin/VFs/blast/blast.cgi), and antimicrobial resistance genes were collected using *CARD* (https://card.mcmaster.ca/analyze/rgi). Plasmid replicon type was determined by using Plasmid Finder 2.1 from the CGE. The conjugal transfer elements were identified using *oriTfinder* web-based tool (https://tool-mml.sjtu.edu.cn/oriTfinder/oriTfinder.html). Comparisons of pMCR, pNDM, and other similar plasmids were analysed using the BLAST Ring Image Generator (BRIG) software (http://sourceforge.net/projects/brig). Since the pMCR or pNDM mapped to many genomes, the top ten genomes were selected based on the BLAST scores to perform genome comparisons. Comparisons of *iuc* and *iro* clusters, and genetic environments of *bla*_NDM−7_ and *mcr1.1* were performed using *GBKviz* (https://gbkviz.streamlit.app/).

### Plasmid conjugation experiment

To investigate the transferabilities of *bla*_NDM−7_, *mcr1.1*, and virulence factors, conjugation experiments were performed between ECO20 and sodium azide-resistant *E. coli* J53. Both donor and recipient isolates were cultured to logarithmic and mixed in equal volumes and incubated at 37℃ overnight to facilitate conjugation. The agar containing sodium azide (80ug/ml) was used to calculate the number of recipients. Colistin (0.5 ug/ml) was used to screen transconjugants containing *mcr1.1*. Meropenem (0.25 ug/ml) was used to screen transconjugants containing *bla*_NDM−7_. Gentamicin (0.5 ug/ml) were used to screen transconjugants containing *iuc* clusters. The agar containing sodium azide and colistin or meropenem or gentamicin were used to screen transconjugants carrying *bla*_NDM−7_ or *mcr-1or iuc*. The presence of *bla*_NDM−7_, *mcr*, and *iuc* in transconjugants was confirmed by PCR and corresponding resistance phenotyping. The primers (*irp3*, *bla*_NDM−7_, *mcr-1*, and *iutA*) were provided in Table S1. Frequencies of conjugation transfer were calculated by the number of transconjugants per recipient. The experiments were repeated five times. S1 nuclease-Pulsed-Field Gel Electrophoresis (S1-PFGE) was further performed to confirm the plasmids in transconjugants. Genomic DNA digested with S1 nuclease was subjected to PFGE as described previously [13].

### Chrome azurol S (CAS) agar plates

Chrome Azurol S (CAS) agar plates were utilized for qualitative detection of siderophore production. To prepare the CAS solution, 60.5 mg of CAS was dissolved in 50 mL of ddH2O. Subsequently, 1 mmol FeCl3·6H2O dissolved in 10 mL of 10 mM hydrochloric acid was added to this solution. After thorough mixing, the resultant mixture was slowly poured into a solution of acetyltrimethylammonium bromide (CTAB) (72.9 mg CTAB dissolved in 40 mL ddH2O), followed by high-pressure sterilization. The CAS-containing King’s B medium was prepared by autoclaving an appropriate amount of King’s B medium agar powder in 700 mL ddH2O. To this medium at a temperature of 50 °C ~ 60 °C, 200 mL of 10×PIPES buffer and 100 mL of the CAS solution were added. This medium was poured slowly onto plates and layered with LB agar that had been sterilized under high pressure to form CAS double-layer plates. The strains were cultured in a low-nutrient broth to a logarithmic phase and the concentration was adjusted to 10^5^ CFU/ml. Subsequently, 1 µL bacterial culture was inoculated onto these plates. The plates were then incubated at 37 °C for 36 to 48 h, and observation was made for the formation of orange-yellow transparent rings.

### Chrome azurol S (CAS) assay

A single fresh colony was inoculated into LB broth and incubated overnight at 37 °C. The following day, a 1:1000 dilution was made from this culture into low-nutrient broth and incubated overnight at 37 °C. Following centrifugation of 1 mL of the culture at 12,000 rpm for 10 min, the supernatant was collected. A CAS detection solution was prepared to achieve a final concentration of 1.2 mM CTAB, 2 mM CAS, and 1 mM FeCl_3_·6H2O. In triplicate, 100 µL of bacterial supernatant was added to a 96-well plate and mixed with an equal volume of the CAS detection solution. This mixture was incubated in darkness for 30 min, and the absorbance was measured at OD630 nm. Optical density values (As) were recorded using sterile low-nutrient broth as a reference (Ar). The siderophore activity unit Su (Siderophore unit) was calculated as [(Ar − As)/Ar]×100 [14]. The optical density values (As) were recorded, using a sterile low-nutrient broth as a reference (Ar). The experiment was repeated thrice. GraphPad Prism 9 (GraphPad Software, San Diego, CA, USA) was used to assess the statistical significance of the data with the student’s t-test and the log-rank test.

## Results

### Identification and resistance phenotypes

Clinical *E. coli* strains (ECO20 and ECO21) were isolated from hospitalized patients at a hospital in Jining, Shandong. Notably, both strains exhibited the presence of both *bla*_NDM−7_ and *mcr-1* genes. ECO20 was recovered from the urine sample of Patient 1 in the intensive care unit on February 17, 2021, while ECO21 was obtained from Patient 2’s sputum in the neurology department on February 19, 2021 (Figure S1). Despite their non-concurrent presence in the same ward, clinical records reveal a compelling temporal and spatial correlation. Patient 2, carrying ECO21, had a prior admission to the ICU ward from February 09 to February 18, 2021 (Figure S1). During an overlapping interval (February 15–18), both Patient 1 and Patient 2 coexisted within the ward, suggesting relevant temporal and spatial proximity influencing transmission dynamics.

Antimicrobial susceptibility testing revealed that both *E. coli* isolates conferred resistance to almost antimicrobials tested, including ampicillin, ampicillin/sulbactam, piperacillin/tazobactam, cefazolin, cefuroxime, ceftriaxone, cefepime, cefoperazone/sulbactam, gentamicin, trimethoprim/sulphamethoxazole, imipenem, meropenem, and colistin, but no resistance to tigecycline, nitrofurantion, and amikacin (Table 1). Thus, these strains exhibited multidrug-resistant profiles.

**Table 1:** The genome characteristics of *Escherichia coli* in this study

Characteristics	Genomes	Accession no.	Length (bp)	GC content (%)	Incompatibility group	Conjugal transfer elements				Resistance factors	Siderophores
						oriT	Relaxase	T4CP	T4SS		
ECO20	Chromosome	CP139891.1	4,794,335	50.56	/^a^	-^b^	-	-	-	-	Yersiniabactin
	pVir	CP139892.1	175,772	50.07	IncFIB/IncFII	+^c^	+	+	+	AAC(3)-IId, dfrA17, TEM-1, sul2, APH(3’’)-Ib, APH(6)-Id, tet(A)	Aerobactin, Salmochelin
	pNDM	CP139893.1	46,161	46.65	IncX3	-	+	+	+	NDM-7	-
	pMCR	CP139894.1	33,309	41.84	IncX4	-	+	+	+	MCR1.1	-
ECO21	Chromosome	CP139887.1	4,795,653	50.56	/	-	-	-	-	-	Yersiniabactin
	pVir	CP139888.1	175,772	50.07	IncFIB/IncFII	+	+	+	+	AAC(3)-IId, dfrA17, TEM-1, sul2, APH(3’’)-Ib, APH(6)-Id, tet(A)	Aerobactin, Salmochelin
	pNDM	CP139889.1	46,161	46.65	IncX3	-	+	+	+	NDM-7	-
	pMCR	CP139890.1	33,309	41.84	IncX4	-	+	+	+	MCR1.1	-

^a^/, not applicable

^b^+, have such information,

^c^-, no such information

### Genomic characteristics

To further investigate the clonal relationship and genomic features, we sequenced and assembled their genomes. Notably, the ECO20 genome shared 99.99% Average Nucleotide Identity (ANI) with ECO21 (Figure S2), indicating the clonal transmission of these two isolates. The two strains belonged to ST4456, fimH20 and H4:O83, and harboring some common virulence genes such as *csgA*, *fdeC*, *hlyF*, *ibeA*, *iss*. They exhibited identical plasmid profiles: a 33 Kb pMCR plasmid, a 46 Kb pNDM plasmid, and a 175 Kb pVir plasmid (Table 2). The pMCR plasmid belonging to the IncX4 type carried the colistin resistance gene *mcr-1*, while the pNDM plasmid belonging to the IncX3 type carried the carbapenem resistance gene *bla*_NDM−7_. The pVir plasmid (IncFIB/IncFII type) harbored aerobactin and salmochelin-associated virulence genes, alongside additional resistance genes, including *AAC(3)-IId, dfrA17, TEM-1, sul2, APH(3’’)-Ib, APH(6)-Id, and tet(A).* Notably, all plasmids possessed conjugal transfer elements, albeit the *oriT* sequences of pNDM and pMCR were not identified by the *oriTfinder* web tool.

**Table 2:** Antimicrobial susceptibilities of strains and their transconjugants

Strains	MIC (µg/mL)^a^																
	AMP	SAM	TZP	CZO	CXM	CRO	FEP	CSL	GEN	LVX	SXT	IPM	MEM	TGC	NIT	AMK	COE
ECO20	≥ 64^R^	≥ 64/32^R^	≥ 256/4^R^	≥ 16^R^	≥ 64^R^	≥ 8^R^	≥ 64^R^	≥ 128/64^R^	≥ 64^R^	2^R^	≥ 8/152^R^	16^R^	16^R^	≤ 0.5^S^	≤ 8^S^	≤ 8^S^	8^R^
ECO21	≥ 64^R^	≥ 64/32^R^	≥ 256/4^R^	≥ 16^R^	≥ 64^R^	≥ 8^R^	≥ 64^R^	≥ 128/64^R^	≥ 64^R^	1^I^	≥ 8/152^R^	32^R^	16^R^	≤ 0.5^S^	≤ 8^S^	≤ 8^S^	8^R^
J53	≤ 2^S^	4/2^S^	≤ 8/4^S^	2^S^	4^S^	≤ 0.25^S^	2^S^	≤ 4/2^S^	≤ 1^S^	≤ 0.5^S^	≤ 0.25/4.75^S^	≤ 0.25^S^	≤ 0.25^S^	≤ 0.5^S^	≤ 16^S^	≤ 2^S^	≤ 0.5^S^
**Transconjugants**																	
T1	8^S^	8/4^S^	≤ 8/4^S^	4^I^	8^S^	≤ 0.25^S^	≤ 2^S^	8/4^S^	≤ 1^S^	≤ 0.5^S^	≤ 0.25/4.75^S^	≤ 0.25^S^	≤ 0.25^S^	≤ 0.5^S^	≤ 8^S^	≤ 8^S^	4^R^
T2	≥ 64^R^	≥ 64/32^R^	≥ 256/4^R^	≥ 16^R^	≥ 64^R^	≥ 8^R^	64^R^	≥ 128/64^R^	≤ 1^S^	≤ 0.5^S^	4/76^S^	32^R^	32^R^	≤ 0.5^S^	≤ 8^S^	≤ 8^S^	≤ 0.5^S^
T3	≥ 64^R^	≥ 64/32^R^	≥ 256/4^R^	≥ 16^R^	≥ 64^R^	≥ 8^R^	≥ 64^R^	≥ 128/64^R^	≤ 1^S^	≤ 0.5^S^	2/38^S^	32^R^	64^R^	≤ 0.5^S^	≤ 8^S^	≤ 8^S^	4^R^
T4	≥ 64^R^	≥ 64/32^R^	≥ 256/4^R^	≥ 16^R^	≥ 64^R^	≥ 8^R^	≥ 64^R^	64/32^R^	16^R^	≤ 0.5^S^	≥ 8/152^R^	16^R^	8^R^	≤ 0.5^S^	≤ 8^S^	≤ 8^S^	4^R^

^a^ AMP, ampicillin; SAM, ampicillin/sulbactam; TZP, piperacillin/tazobactam; CZO, cefazolin; CXM, cefuroxime; CRO, ceftriaxone; FEP, cefepime; CSL, cefoperazone/sulbactam; GEN, gentamicin; LVX, levofloxacin; SXT, trimethoprim/sulphamethoxazole; IPM, imipenem; MEM, meropenem; TGC, tigecycline; NIT, nitrofurantion.AMK, amikacin; COE, colistin

### Transferability of resistance and virulence plasmids

**Fig. 1:**
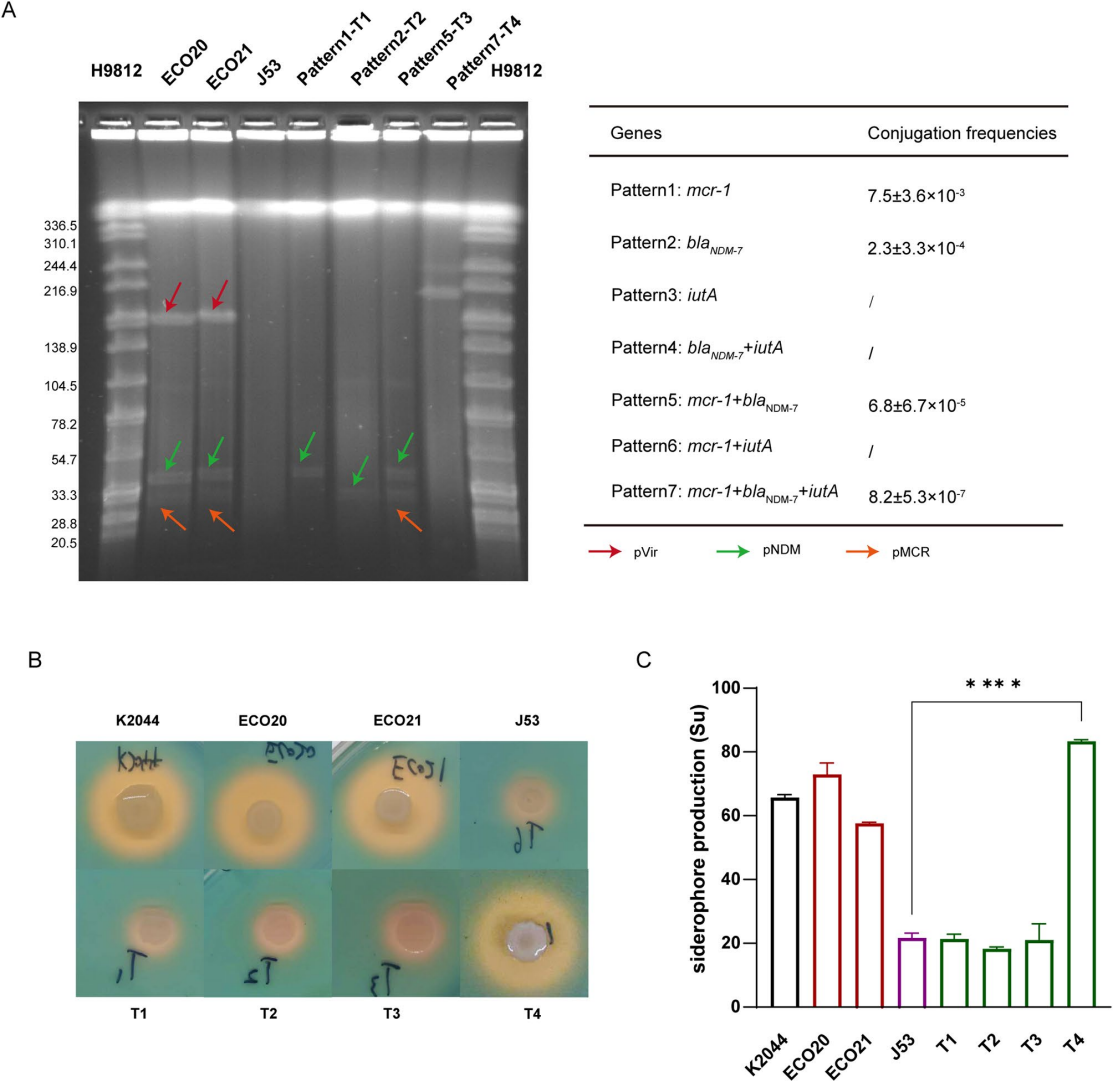
Transmission of pMDR, pNDM, and pVir plasmids. (**A**) Plasmid profiles of transconjugants and transfer patterns. (**B**) Siderophore production by CAS plates. K1-type *Klebsiella pneumoniae* NTUH-K2044 (K2044) was used as a positive control strain. (**C**) Siderophore production by CAS liquid assays

To investigate the transmissibility of *mcr-1*, *bla*_NDM−7_, and *iutA*, we subjected ECO20 to conjugation experiments with *E. coli* J53. As shown in Fig. 1A, there should be seven plasmid conjugation patterns. However, after many conjugation experiments, we only observed four different transfer patterns (Fig. 1A). Importantly, the co-transfer of *mcr-1*, *bla*_NDM−7_, and *iutA* led to the formation of a fusion plasmid (Fig. 1A), suggesting that diverse recombination events would occur during the plasmid conjugation process. High conjugation frequencies of *mcr-1* and *bla*_NDM−7_ indicate broad spread and effective transfer of pMCR and pNDM plasmids (Fig. 1A). The transfer of resistance genes *mcr-1* and *bla*_NDM−7_ conferred their corresponding resistance phenotypes against colistin and carbapenem to *E. coli* J53 (Table 1). Similarly, virulence genes with their corresponding virulence phenotypes characterized by high siderophore production were also successfully transferred to *E. coli* J53. The transconjugant T4 which acquired the pVir plasmid from ECO20 showed significantly higher siderophore production than J53 (Fig. 1B and C).

### Prevalence of pMCR and pNDM plasmids

**Fig. 2:**
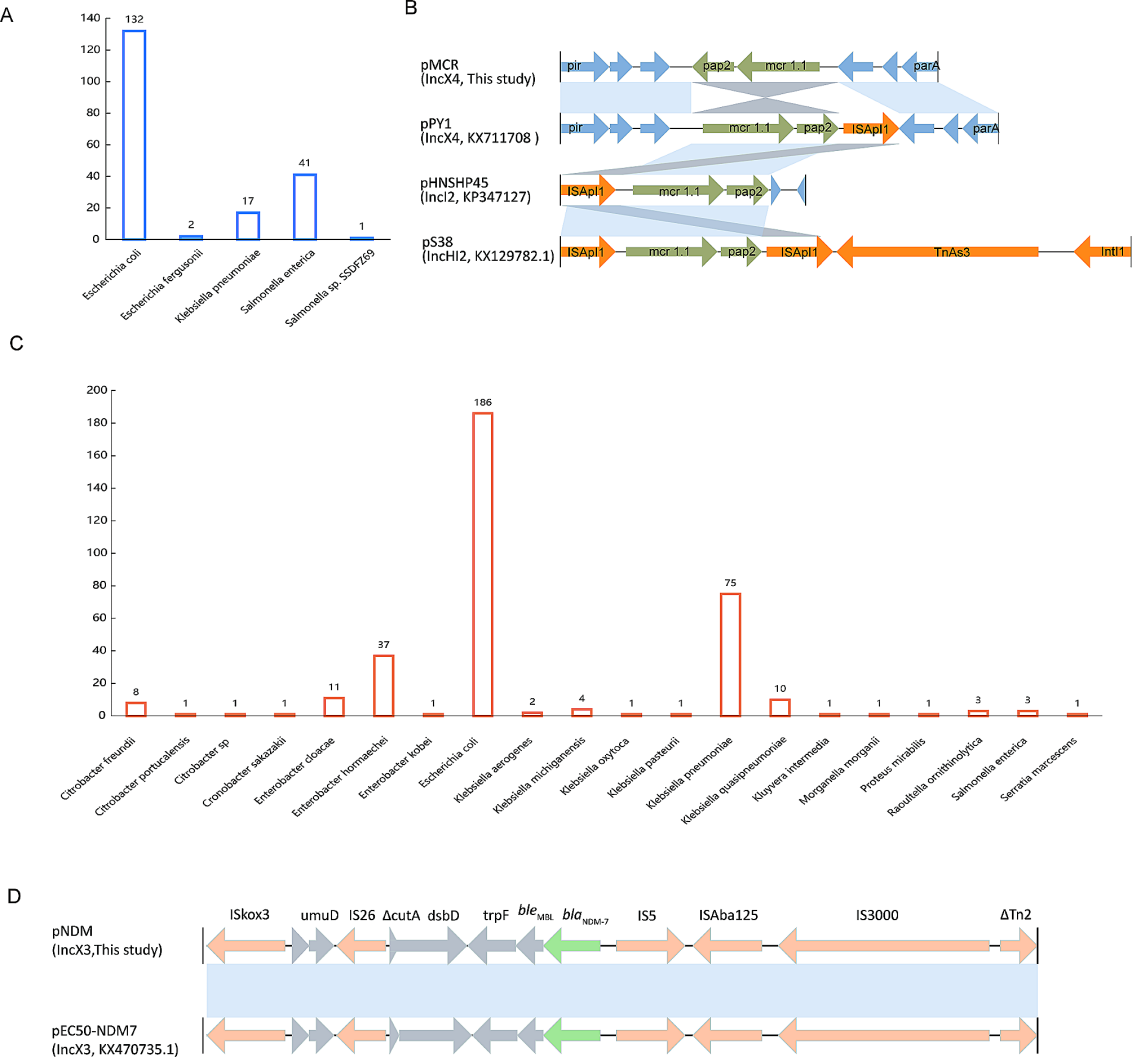
Occurrence and genetic environments of IncX4-type-pMCR and IncX3-type pNDM plasmids from NCBI database. (**A**) Occurrence of IncX4-type-pMCR plasmids across species. (**B**) Genetic environment of *mcr1.1*. (**C**) Occurrence of IncX3-type pNDM plasmids across species. (**D**) Genetic environment of *bla*_NDM−7_

Blastn analysis revealed that the IncX4-type pMCR plasmid closely resembled numerous plasmids previously submitted to the NCBI database (Figure S3). These similar plasmids exhibited >99% coverages and >99% identities. Interestingly, it was not confined to *E. coli* but was also found in *Klebsiella* and *Salmonella* (Fig. 2A). Moreover, the single-ended Tn*6330* variant (IS*Apl1*-*mcr1.1-pap2*) is commonly present in *mcr-1.1*-harbouring plasmids, such as pHNSHP45(IncI2, KP347127) and pS38(IncHI2, KX129782.1), despite that IS*Apl1* was downstream of *mcr1.1* and *pap2* in pPY1(IncX4, KX711708) (Fig. 2B). In contrast, pMCR in this study had a different genetic environment for *mcr1.1* with the *pap2* located downstream and IS*Apl1* deleted (Fig. 2B).

The pNDM-like IncX3 plasmid was also identical to many plasmids from the NCBI database (Figure S4). These similar plasmids had 100 coverages and > 99% identities. They were frequently observed in gram-negative bacteria, exhibiting a broader host range compared to the pMCR-like IncX4. Apart from *E. coli* and *Klebsiella*, the pNDM-like IncX3 plasmid was also detected in *Enterobacter, Citrobacter*, and various other bacterial species (Fig. 2C). Nonetheless, *E. coli* was still the primary host bacterium carrying the *bla*_NDM−7_-positive IncX3 plasmid (Fig. 2C). Unlike the prevalence observed in pMCR-like IncX4, the incidence of pNDM-like IncX3 plasmids was lower in *Salmonella* (Fig. 2C). Furthermore, *bla*_NDM−7_ was associated with the complex transposon structure *(*IS*26-*IS*3-*IS*Aba125-*IS*5-bla*_NDM−7_*- ble*_*MBL*_*-trpF-dsbD-*IS*26)* which is also commonly present in other *bla*_NDM−7_-positive plasmids and plasmids carrying NDM variants (Fig. 2D).

### Characteristics of the IncFIB_K_/IncFII virulence plasmid

**Fig. 3:**
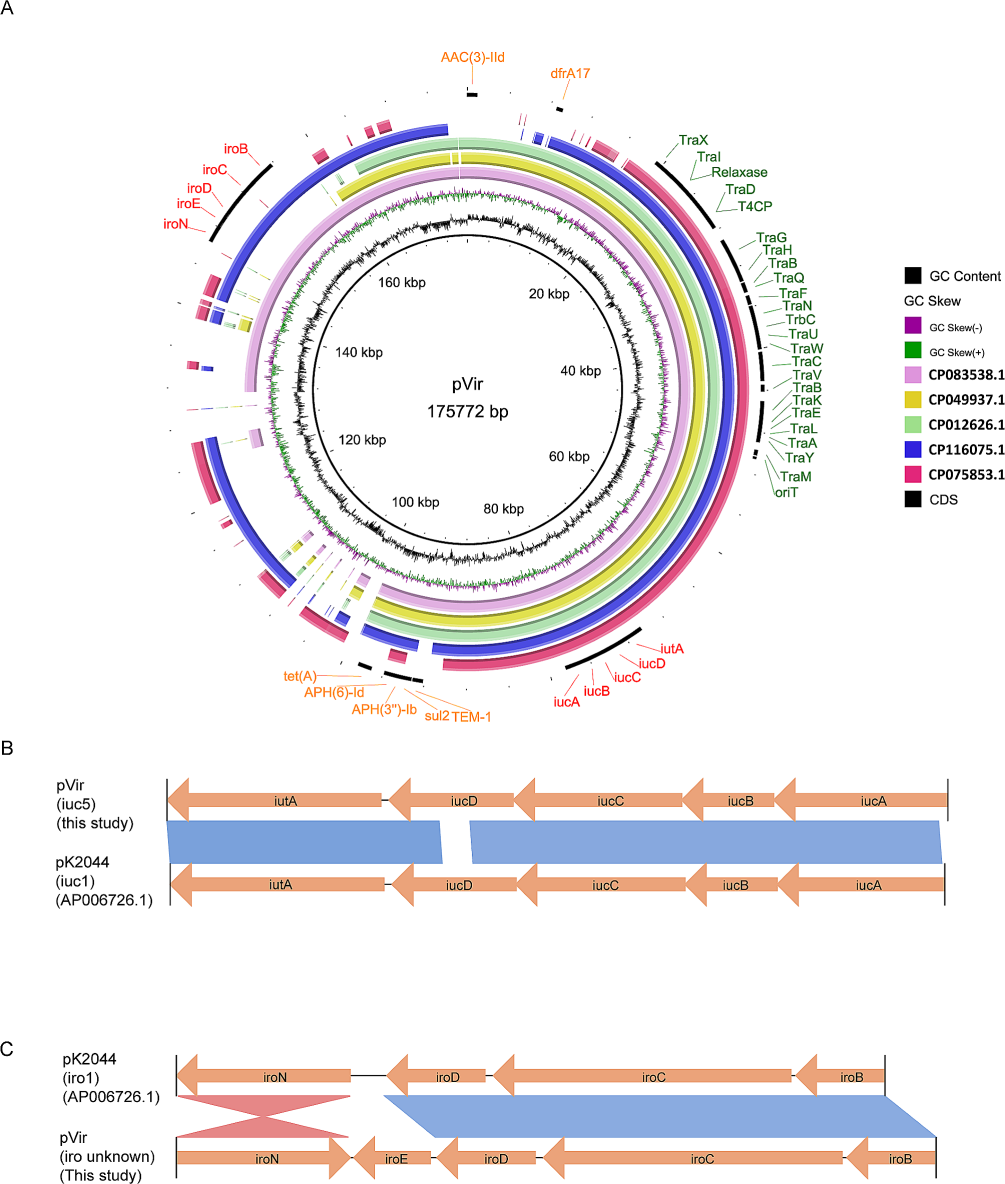
Characteristics of siderophores-encoding pVir plasmids. (**A**) Comparative analysis of pVir plasmids and some similar plasmids from NCBI database. The reference plasmid is pVir (CP139892.1). (**B**) Comparisons of iuc and iro clusters carried by *E. coli* pVir plasmid and Klebsiella pneumoniae pK2044 plasmid

The 175,772 bp pVir plasmid contained virulence and resistance genes, along with a Type IV Secretion System (T4SS) (Fig. 3A). The aerobactin genes (*iucABCD-iutA*) and salmochelin genes (*iroBCDEN*) endowed it with high siderophore production (Fig. 1B and C). Notably, the schematic structure of *iuc* clusters (*iuc1*) was similar to that of virulence plasmid pK2044 in *Klebsiella* (*iuc1*) (Fig. 3B), while the *iro* clusters in pVir had *iroE* added and *iroN* inversion compared to pK2044 (Fig. 3C). The complete conjugal transfer element T4SS conferred the ability of self-transfer (Fig. 2A), and some resistance genes conferred resistance to aminoglycosides, cephalosporins, sulfonamides, tetracyclines, and diaminopyrimidines (Table 1). No identical plasmid was found in the NCBI nucleotide database. Some similar plasmids were selected to perform comparative genome analysis with pVir in this study. Most plasmids cannot possess all the above elements for self-transfer, resistance, and siderophore production, except for the unnamed plasmid (CP083538) (Fig. 3A).

## Discussion

The escalating threat of multidrug-resistant *Enterobacterales* (*MDRE*) represents a profound challenge in healthcare settings worldwide [15]. Our investigation highlights the emergence of carbapenemase genes (such as *bla*_NDM_) and the plasmid-mediated colistin resistance gene (*mcr-1*) within clinical *E. coli* strains. The coexistence of these resistance determinants, along with the concurrent presence of siderophore-producing plasmids, indicates a worrisome confluence that could lead to the emergence of multidrug-resistant and highly pathogenic pathogens.

The isolation of genetically identical strains (ECO20 and ECO21) carrying identical plasmid profiles from patients in distinct wards raises critical concerns about nosocomial transmission. The spatial and temporal correlation between patients suggests a possible route of transmission within the hospital environment. Notably, these strains exhibited extensive resistance profiles against a range of antibiotics, especially carbapenems and colistin, limiting treatment options in clinical settings. These findings resonate with the global trend in the dissemination of carbapenemases and colistin resistance in *E. coli*, as documented by recent studies [1, 4]. In contrast to NDM-1, NDM-7 has two amino acid substitutions (D130N and M15L) and is more active as a carbapenemase [16]. Its dissemination, mainly associated with the IncX3 conjugative plasmid [17], shares a genetic environment with most NDM variants, notably the IS26-flanked pseudo-composite transposons (IS*26-*IS*3-*IS*Aba125-*IS*5-bla*_NDM_*-ble*_MBL_*-trpF-dsbD-*IS*26*) [18], which likely represents the most important contributor to the widespread of *bla*_NDM_ genes. Our study finds that the IncX3-type pNDM plasmids, while observed across diverse gram-negative bacteria, exhibit preferential colonization in certain species, such as *E. coli* and *Klebsiella*. Their ability to traverse diverse ecological niches poses a significant public health threat.

The *mcr* gene exerts colistin resistance by encoding a phosphoethanolamine transferase and can be located on various plasmids, including the IncI2, IncX4, IncP, IncY, IncFII, and IncHI2 plasmid incompatibility clusters [19], which are considered to be important vectors for facilitating the spread of *mcr-1*. Among these, the most common plasmid types are IncI2, IncHI2, and IncX4 [19]. The *mcr-1* in this study was located on conjugative IncX4 plasmids. While IncX4-type pMCR plasmids were found in a narrower range of bacterial species compared to IncX3-type pNDM plasmids, their prevalence was notably higher in *Salmonella*. This highlights distinct species-specific dissemination patterns observed for these resistance plasmids. The single-ended Tn*6330* variant (IS*Apl1-mcr1.1-pap2*) is commonly present in *mcr1.1*-harbouring plasmids [20], which drives the dissemination of *mcr1.1* genes among various bacterial species. Even though plasmids can spread rapidly within species and across geographical regions, they aren’t very likely to establish themselves in new bacterial environments. Such exchanges, however, provide opportunities for between-plasmid transposon jumps and genetic recombination, which significantly contribute to the spread of antimicrobial resistance genes.

The co-occurrence of *bla*_NDM_-positive plasmid and *mcr*-positive plasmid possibly occurred, and several studies have been reported [21–23]. However, the co-existence of *bla*_NDM−7_ positive IncX3 plasmid and *mcr1.1*-positve IncX4 plasmid in clinical *E. coli* isolates which we described in this study was previously underreported. The IncX3 and IncX4 were the most common plasmids carrying *bla*_NDM_ and *mcr*, respectively [17, 19], thus, plasmid transfer and evolution may allow the co-transfer of *bla*_NDM_ and *mcr* to become prevalent and spread in the future.

Additionally, our study highlights the presence of a 175 kb pVir plasmid in ECO20 and ECO21, mediating high siderophore production via aerobactin and salmochelin encoding high-affinity siderophores (aerobactin and salmochelin) which are considered as critical virulence factors enabling bacteria to compete for iron with the host [9]. Adequate iron is crucial for bacteria to survive in limited environments [24]. The existing study focuses more on the siderophore-production *K. pneumoniae* for the rapid emergence of multidrug-resistant hypervirulent *K. pneumoniae* has attracted widespread attention [25, 26]. However, the importance of siderophore production extends beyond *Klebsiella* to encompass all pathogens. Notably, the *iuc* and *iro* clusters differed from those found in hypervirulent *K. pneumoniae*, suggesting a distinct ancestral background. The absence of identical plasmids in existing databases accentuates the novelty and significance of pVir, indicating its potential role in driving the evolution of highly adaptable and pathogenic strains.

Our results elucidate the conjugative abilities of *bla*_NDM−7_-positive IncX3 plasmid, *mcr1.1*-positive IncX4 plasmid, and *iuc*-positive IncFIB/IncFII plasmid, and describe four transfer patterns. Interestingly, the formation of fusion plasmid illustrates the potential genetic recombination during plasmid conjugation. Importantly, the transfer of resistance and virulence genes endows bacteria with corresponding resistance and virulence phenotypes, indicating its potential role in driving the evolution of highly adaptable and pathogenic strains. This emphasizes the urgency to prevent the spread of these plasmids and associated genes.

However, our study has limitations. The analysis was limited to two clinical isolates from a specific geographical location, restricting the generalizability of our findings. Moreover, the study has not detected all possible plasmid transfer patterns. Further studies involving a broader spectrum of isolates from diverse geographic regions are crucial to elucidate the extent of plasmid dissemination and genetic diversity.

Future research should delve into the intricate mechanisms underlying plasmid transfer and recombination, deciphering the molecular determinants governing these processes. Moreover, surveillance and control measures within healthcare settings are imperative to curb the spread of *MDR E. coli* strains and prevent the emergence of highly resistant and virulent strains. In conclusion, our study underscores the urgency of understanding the dynamics of plasmid-mediated resistance and virulence, emphasizing the need for robust surveillance and intervention strategies to mitigate the escalating threat posed by *MDRE* strains.

### Electronic supplementary material

Below is the link to the electronic supplementary material.

Supplementary Material 1

Supplementary Material 2

## Data Availability

The nucleotide sequences of the chromosome and plasmids of E. coli isolates ECO20 and ECO21 have been deposited in the NCBI database (BioProject accession number PRJNA1005813).
