# Supplementary material for: Dissemination of clinical Escherichia coli strains harboring mcr-1, blaNDM−7 and siderophore-producing plasmids in a Chinese hospital
Source: Antimicrob Resist Infect Control. 2024 Jun 18;13:66. doi: 10.1186/s13756-024-01423-3 (PMC11184858; doi:10.1186/s13756-024-01423-3)
Supplement: Supplementary file 1 — Supplementary Material 1 [file 13756_2024_1423_MOESM1_ESM.docx]

**Supplementary materials**

**
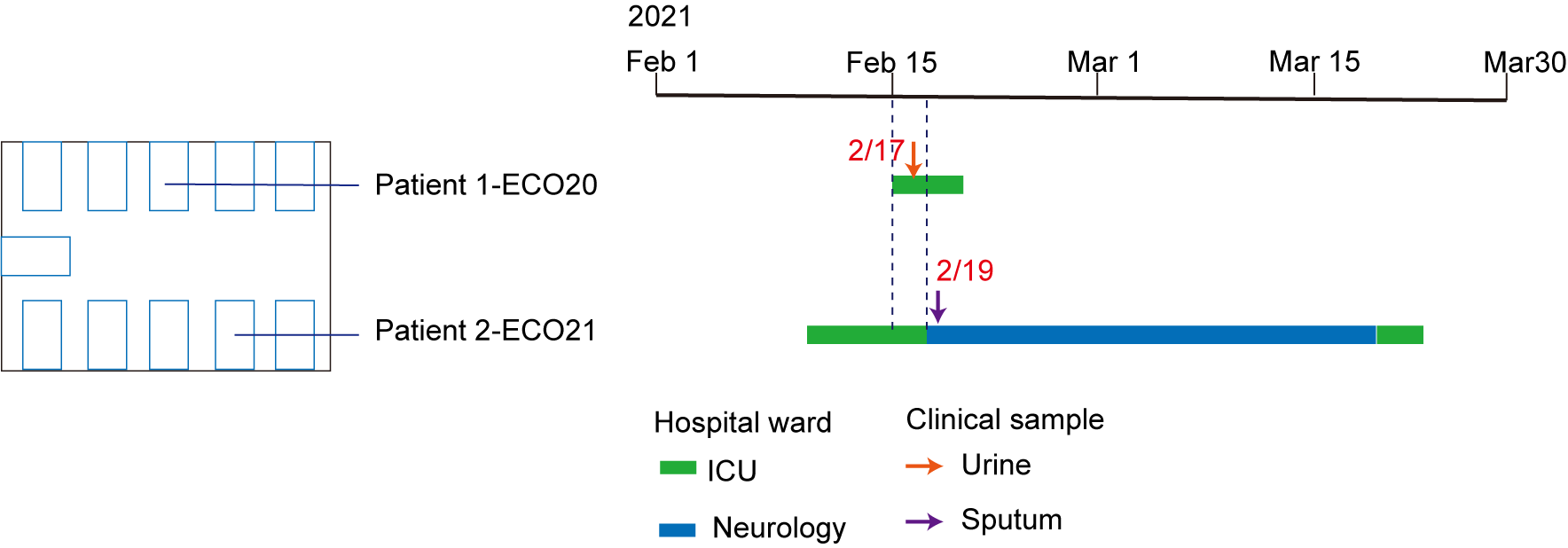
**

**Fig. S1 Timeline of the detection of ECO20 and ECO21.** Coloured rectangles represent the presence of the patient in the corresponding ward. The coloured arrows represent the detection of strains corresponding clinical samples.

**
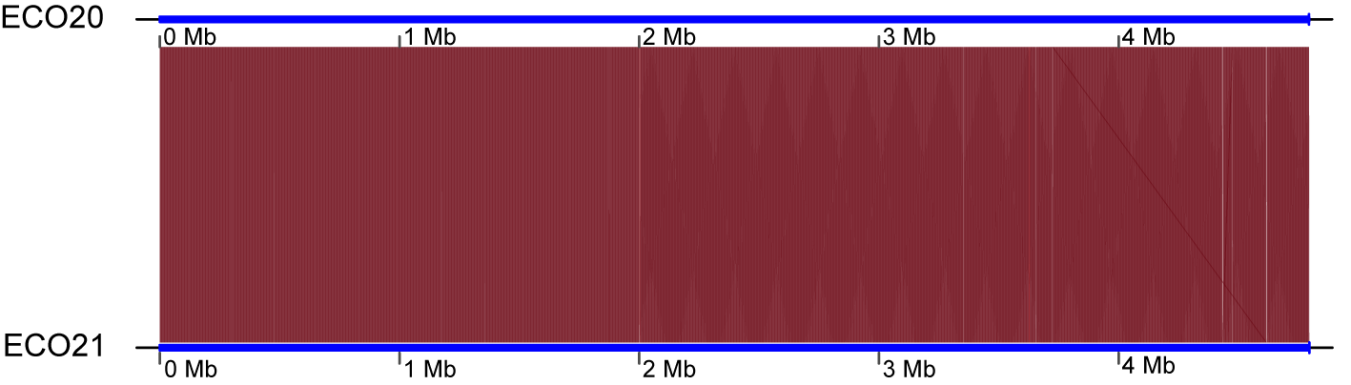
**

**Fig. S2 The ANI of ECO20 genome comparing with ECO21genome.**

**
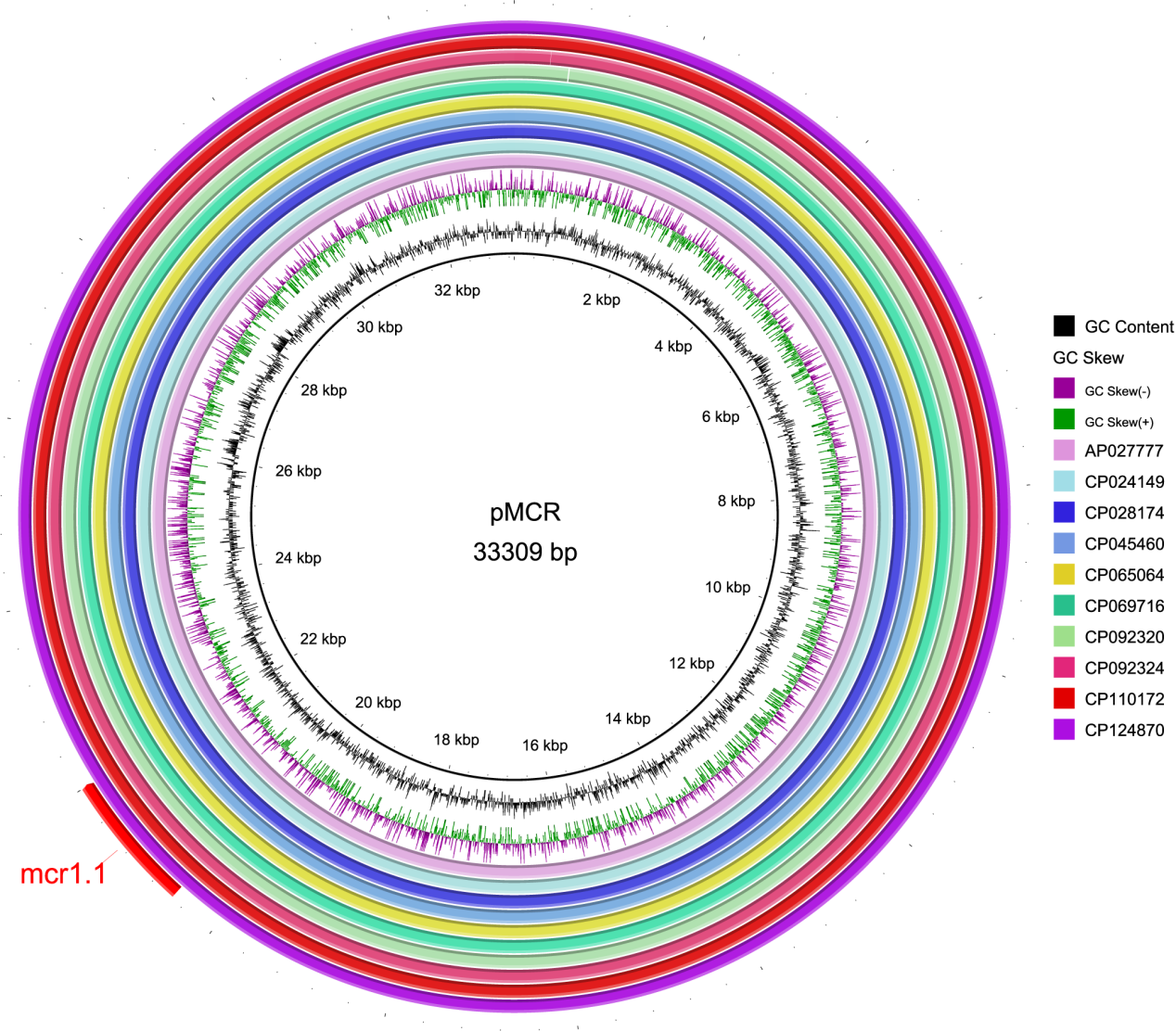
Fig. S3 Comparative analysis of pMCR and some similar plasmids from NCBI database.**

**
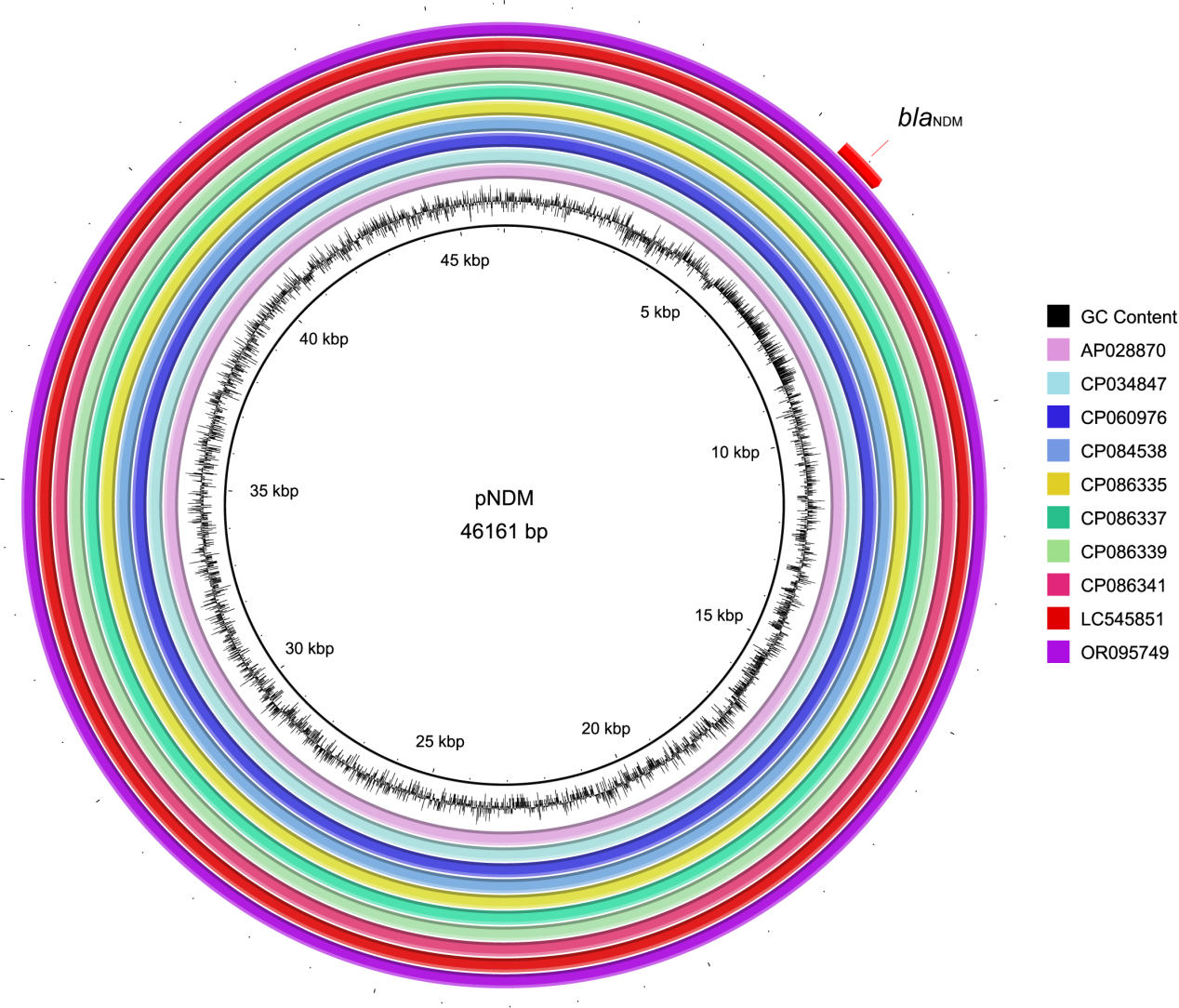
Fig. S4 Comparative analyisis of pNDM and some similar plasmids from NCBI database.**

**Table S1 Primers used in this study**

**Table S1 Primers used in this study**

| **Name** | **Sequences (5’-3’)** |
| --- | --- |
| **For screening transconjugants** | |
| KPC-F | AGGACTTTGGCGGCTCCAT |
| KPC-R | TCCCTCGAGCGCGAGTCTA |
| NDM-F | GTTTGGCGATCTGGTTTTC |
| NDM-R | CGGAATGGCTCATCACGATC |
| OXA-48-F | GCGTGGTTAAGGATGAACAC |
| OXA48-R | CATCAAGTTCAACCCAACCG |
| IMP-F | GGAATAGAGTGGCTTAAYTCTC |
| IMP-R | GGTTTAAYAAAACAACCACC |
| SPM-F | AAAATCTGGGTACGCAAACG |
| SPM-R | ACATTATCCGCTGGAACAGG |
| VIM-F | GATGGTGTTTGGTCGCATA |
| VIM-R | CGAATGCGCAGCACCAG |
| MCR-F | CGGTCAGTCCGTTTGTTC |
| MCR-R | CTTGGTCGGTCTGTAGGG |
| iutA-F | GGGAAAGGCTTCTCTGCCAT |
| iutA-R | TTATTCGCCACCACGCTCTT |
| Irp3-F | GGGCTGCTGCTATTGGGTAA |
| Irp3-R | CTGGATGACCAGATGACGGG |
