# Supplementary figures and images for: Dissemination of clinical Escherichia coli strains harboring mcr-1, blaNDM−7 and siderophore-producing plasmids in a Chinese hospital
Source: Antimicrob Resist Infect Control. 2024 Jun 18;13:66. doi: 10.1186/s13756-024-01423-3 (PMC11184858; doi:10.1186/s13756-024-01423-3)

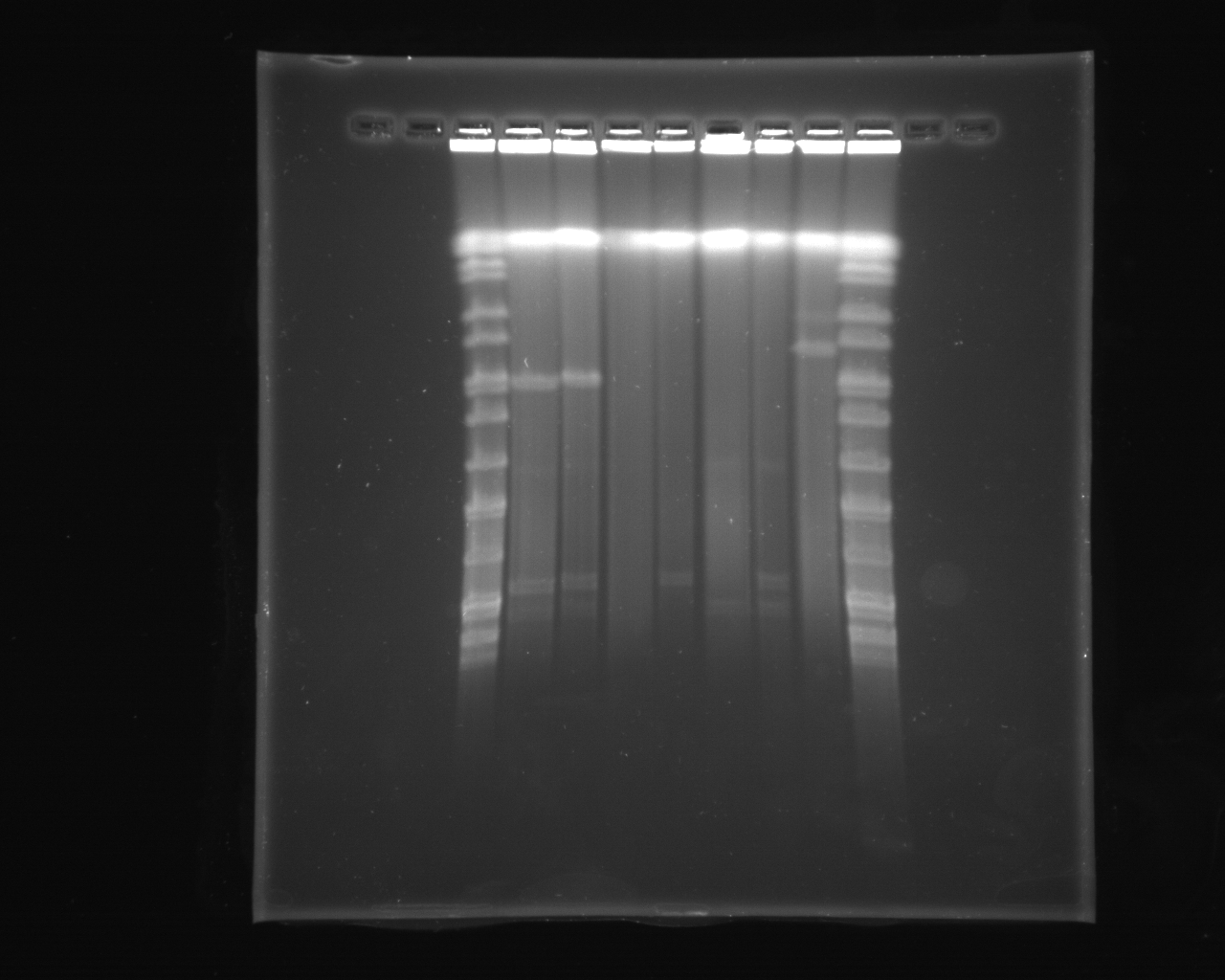

Supplement: Supplementary file 2 — Supplementary Material 2 [file 13756_2024_1423_MOESM2_ESM.tif]
